# A Miniaturized MicroRNA Sensor Identifies Targets Associated with Weight Loss in a Diet and Exercise Intervention among Healthy Overweight Individuals

**DOI:** 10.3390/s22186758

**Published:** 2022-09-07

**Authors:** Vidura Jayasooriya, Nathaniel Johnson, Adam Bradley, Christopher Kotarsky, Lizzy Jepng’etich, Daniel Friesner, Sherri Stastny, Kyle J. Hackney, Dharmakeerthi Nawarathna

**Affiliations:** 1 Department of Electrical and Computer Engineering, North Dakota State University, Fargo, ND 58108, USA; 2 Department of Health, Nutrition, and Exercise Sciences, North Dakota State University, Fargo, ND 58108, USA; 3 Department of Statistics, North Dakota State University, Fargo, ND 58108, USA; 4 Department of Pharmacy Practice, North Dakota State University, Fargo, ND 58108, USA

**Keywords:** microRNA, weight loss interventions, electric fields, dielectrophoresis, point-of-care

## Abstract

Weight loss through dietary and exercise intervention is commonly prescribed but is not effective for all individuals. Recent studies have demonstrated that circulating microRNA (miR) biomarkers could potentially be used to identify individuals who will likely lose weight through diet and exercise and attain a healthy body weight. However, accurate detection of miRs in clinical samples is difficult, error-prone, and expensive. To address this issue, we recently developed *iLluminate*—a low-cost and highly sensitive miR sensor suitable for point-of-care testing. To investigate if miR testing and *iLluminate* can be used in real-world obesity applications, we developed a pilot diet and exercise intervention and utilized *iLluminate* to evaluate miR biomarkers. We evaluated the expression of miRs-140, -935, -let-7b, and -99a, which are biomarkers for fat loss, energy metabolism, and adipogenic differentiation. Responders lost more total mass, tissue mass, and fat mass than non-responders. miRs-140, -935, -let-7b, and -99a, collectively accounted for 6.9% and 8.8% of the explained variability in fat and lean mass, respectively. At the level of the individual coefficients, miRs-140 and -935 were significantly associated with fat loss. Collectively, miRs-140 and -935 provide an additional degree of predictive capability in body mass and fat mass alternations.

## 1. Introduction

The rates of being overweight, obese, or severely obese in the United States (US) are ~36%, ~42%, and ~9%, respectively [1,2]. Those living with obesity are at greater risk for type 2 diabetes, hypertension, dyslipidemia, cardiovascular disease, and mortality from several cancers [3]. In addition to individual health consequences, the total health care cost of obesity in the US was estimated at $480.7 billion in 2016 with an additional $1.24 trillion in lost productivity and other indirect costs [4]. However, the high cost, risk of future health problems, and body mass index (BMI) cut-point requirements (>40 or BMI of >35 kg/m^2^) exclude bariatric surgery as the best option for obese patients [5,6]. In contrast, a combination of diet and exercise is commonly used for weight loss interventions with total fat mass loss reduction being achieved through the systematic reduction of dietary energy and increased energy expenditure [7]. However, there can be inter-individual variability in losing body mass, which may leave patients frustrated with this approach [8,9,10]. For example, some individuals start losing weight at the beginning of the intervention but systematically gain weight after the initial success. Others reach a weight loss plateau with the continuation of diet and weight loss intervention [8,10]. Unfortunately, there are no diagnostic tests available to predict the expected outcome of the diet and weight loss intervention of the participants. Thus, a critical roadblock for mitigating obesity is the inability to identify individuals who will succeed with a diet and exercise intervention. Our long-term goal is to introduce point-of-care biomarker testing to identify individuals who will lose weight through diet and exercise.

Recent studies have shown that there is a significant variability in response to weight-loss interventions; this variability is, in part, attributable to epigenetic factors [8,9,10]. These epigenetic factors can be monitored and predicted by evaluating circulating microRNAs (miRs). miRs impact many biological responses in humans and have been utilized as biomarkers for health and disease detection and monitoring [11]. For example, miRs have been connected to various aspects of exercise performance and body composition with distinct changes in several miRs expressions [12,13,14,15,16,17]. Fitness level can influence specific miRs levels [18,19,20,21], and weight loss induced by diet and exercise, or surgery, can impact the expression of multiple miRs [22,23,24,25,26,27,28]. For example, a recent 16 week diet and exercise intervention demonstrated significant variability of miRs-140 and -935 in the individuals who lost at least 5% of body weight (called responders to the intervention) [10]. This evidence shows that miRs could be a viable target to monitor the effectiveness of weight management by diet and exercise. Ideally, there are two fundamental problems to solve before introducing miR testing to weight loss interventions. First, there must be a sensitive miR panel and a point-of-care device that can detect the target miR levels with high sensitivity and low cost. To be effective in weight loss applications, this miR panel should be able to identify the responders in a short (~6 weeks or less) diet and exercise intervention. Second, a study is needed to determine if miR biomarkers utilized in the short intervention can predict the future outcome (continuous weight loss and attaining healthy body weight) if the subject continued the intervention. After completing both studies, miR biomarkers and the detection device can be utilized in real-world diet and exercise interventions. Ideally, subjects will go through a short diet and exercise intervention (6 weeks or less) called the screening phase followed by evaluation of miR biomarker levels. Based on the changes in the miR levels, predictions can be made regarding the subject’s potential to lose weight and attain healthy weight if they continue the intervention. The screening phase should be short because adhering to long (e.g., 16 week) interventions to find the suitability of diet and weight loss is difficult.

Currently, real time quantitative reverse transcriptase polymerase chain reaction (qRT-PCR) is the gold standard for miR detection in blood and serum [29]. However, qRT-PCR can be unreliable for detecting short molecules such as miRs, and it can produce inaccurate results. Clinical qRT-PCR assays are performed in centralized facilities, which are expensive (~$2000 per sample) and time-consuming [30]. It typically takes hours to days to return results; thus, these assays are not suitable for routine testing. There are other miRNA detection techniques such as microarrays, electrochemical, plasmonic, and hybridization-based sensors [31,32,33,34]. At a fundamental level, these techniques use diffusion or a combination of diffusion and sample flow to apply target miRNAs to detection electrodes, or to complementary capture molecules typically immobilized on solid surfaces or electrodes. Diffusion is not a selective or steady state process; thus, it produces results with a large degree of inter-sample variability—especially when used to detect very small quantities (<picomolar levels). In addition, molecular crowding near the detection electrodes produces steric hindrance, which affects the sensitivity, limit of detection, and sensing throughput. To minimize molecular crowding, a significant dilution of serum is needed; typically, studies used ~100 μL serum, diluted ~100x, and a small portion of the diluted sample (<1 mL) is then used in the experiment.

**Figure 1:**
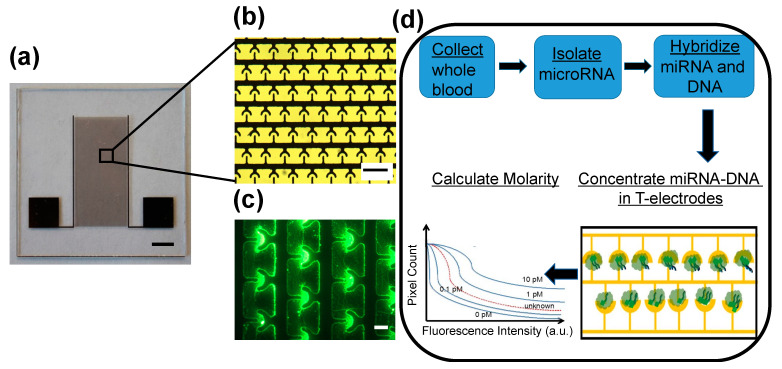
Details of the miR detection device (*iLluminate*) used in the experiments. Device design, fabrication, initial results of miRNA detection, and comparisons of detection, with current gold standard (qRT-PCR) was discussed elsewhere: (**a**) a picture of *iLluminate* device used in experiments. Scale bar shows 5 mm; (**b**) bright field image of interdigitated T-electrodes in the *iLluminate*. The unique shape of T-electrodes needed for efficient concentration of fluorophore labeled target miRs. Scale bar shows 200 µm; (**c**) fluorescence image of the T-electrodes after concentrating let-7b miRNA-DNA (1mM) applying 10 Vpp with 1 MHz electric potential for about 10 min. The circle drawn in broken lines show the T-electrode area. Scale bar shows 100 µm; (**d**) illustrate the steps of miR detection assay using *iLluminate*.

We recently developed a non-enzymatic miR sensing technology called *iLluminate* that utilizes nano-plasmonic and dielectrophoretic, thermophoretic, and electrothermal effects [35]. *iLluminate* is a promising tool for point-of-care settings because there is no complicated machinery required, and integrated detection can be done within 10–15 min. Figure 1a shows a photograph of the device. Briefly, the *iLluminate* device was manufactured on a glass substrate using photolithography, metal deposition, and lift-off techniques. Details of the manufacturing can be found elsewhere [35]. The sample is pipetted on the electrode area. *iLluminate* utilizes interdigitated T-electrodes for efficient concentration of fluorophore-labeled target miRs followed by fluorescence enhancement, recording of the fluorescence intensity, and determination of the molarity. Figure 1b shows a picture of T-electrodes. Briefly, miRNA (target and non-target) is isolated from the serum using a commercially available kit and suspended in a buffer (Qiagen, Germantown, MD, USA). The target miRNA is then hybridized with a fluorescently labeled complementary 22-mer DNA molecules. The hybridization temperature and time are optimized to minimize off-target hybridization and maximize target hybridization. The sample, including hybridized fluorophore-labeled miRNA-DNA and free fluorophore-labelled DNA, is then pipetted to *iLluminate* for detection.

First, a low power electric potential (10 Vpp and 1 MHz) was applied to electrodes to selectively concentrate target fluorophore-labeled miRNA-DNA in the nano-plasmonic hotspot (or hotspots). Hotspots were fabricated in the peripheries of microelectrodes. A 1-MHz electric signal was needed to selectively concentrate fluorophore-labeled miRNA-DNA duplex molecules in the hotspots. We found that free fluorophore-labeled DNA molecules do not concentrate in the hotspots at 1 MHz [35]. Furthermore, earlier, we demonstrated that thermophoresis, electrothermal flow, and dielectrophoretic force, combine to drive fluorophore-labeled miRNA-DNA molecules toward electrodes and concentrate on hotspots [35]. Figure 1c shows a fluorescence image of concentrated fluorescein labeled let-7b miR-DNA molecules in T-electrodes. Once miRNA-DNA molecules are concentrated on peripheries of the electrodes where there are hotspots, the hotspots enhance the fluorescence intensity of the miRNA-DNA molecules via metal-enhanced fluorescence. In our earlier study, we showed that metal-enhanced fluorescence increased the fluorescence by about 100-fold and produced bright fluorescence spots for miRNA-DNA leading to sensitive and specific detection of miRNA [35]. Finally, a fluorescence image of the sample was recorded, and the number of pixels versus fluorescence intensity was calculated and the molarity of each miRNA target was determined by comparing the sample curve with a standard curve of known molarities. Figure 1d illustrates the steps of the miR detection, including miR isolation, hybridization, and detection. Further details about miR detection using *iLluminate* are included in the materials and methods section. Our previous study demonstrated that *iLluminate* has superior performance than qRT-PCR, which is the current gold standard for nucleic acid detection [35]. The objective of this study is two-fold. First, we demonstrate that *iLluminate* can detect changes in potential miR biomarkers related to combined diet and exercise in clinical samples. Second, we study correlations of miR biomarkers and weight loss.

Parr et al. developed a 16 week diet and exercise intervention on 89 male/female obese or overweight subjects [10]. Each subject was exposed to total of 500 kcal/d energy restriction: 250 kcal/d from diet and 250 kcal/d from exercise. Resting plasma samples were collected after an overnight fast pre- and post-intervention. Thirteen miR targets were analyzed using a 96-well miScript miRNA PCR array. The selection of miRs was based on previous studies showing a change in miRNA due to exercise, energy restrictions, or surgical interventions [10]. Four miRs targets (miR-935, -140, -221, and -223) have been identified as potential biomarkers for diet and exercise interventions. The detection of multiple miRs is not feasible in clinical weight-loss interventions, and thus we pre-selected two miRs (miR-935 and -140) for our study. The selection is motivated by evidence showing that miR-140 targets the integral membrane protein called fibronectin type III domain-containing protein 5, secreted by skeletal muscle cells in response to exercise. miR-935 is related to exercise-mediated adaptations and energy metabolism [10]. In addition, we have selected miR-let-7b and 99a as additional biomarkers.

Both miR-let-7b and 99a are related to preadipocytes—cells found in connective tissue and responsible for storage of fat [36,37]. More specifically, Kajimoto et al. reported that both miR-let 7b and -99a are upregulated during the adipogenic differentiation, but these changes were not observed at early time points (days 1–5) [38]. These miRs modulate the function of adipocytes. Therefore, we expect differential expression of one or both miR-let 7b and -99a during the proposed diet and exercise intervention. Frost and Olson demonstrated that the miR-let 7 family, including miR-let 7b, is responsible for impaired glucose intolerance [39]. Studies using the inhibition of the let-7 family with anti-miR could treat or prevent impaired glucose tolerance in mice with diet-induced obesity [39]. This evidence clearly shows the link between impaired glucose tolerance and miR-let7b. Furthermore, obesity is a risk factor for developing diabetes and impaired glucose tolerance. Therefore, we included miR-let-7b and 99a in our study.

## 2. Materials and Methods

### 2.1. Participants

**Figure 2:**
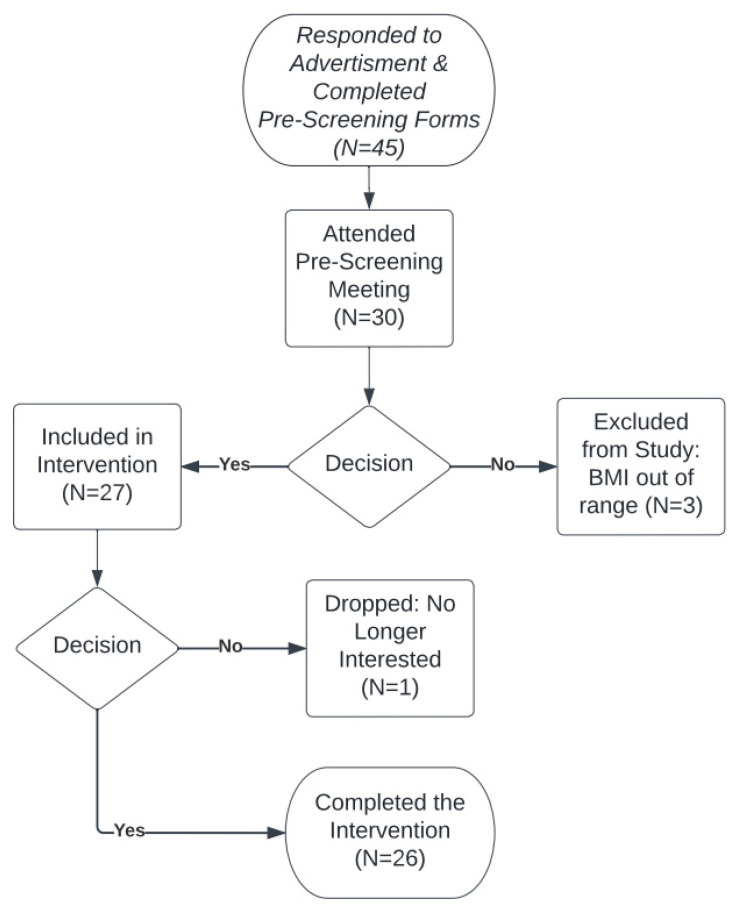
Design of the diet and exercise intervention. Flowchart illustrates the steps of the intervention and number of participants completed each step. Total of 45 subjects were intended to participate but only 26 subjects completed the intervention. Blood samples were collected from 27 subjects and used in pre-intervention miR analysis, and post microRNA was analyzed from 26 subjects who successfully completed the intervention.

Figure 2 illustrates the flowchart for study participation. Forty-five individuals between 18 and 60 years of age were recruited using e-mail, printed advertisement, or word-of-mouth for an informational session. During this session, participants completed both the Physical Activity Readiness Questionnaire+ [30] and a broader health history questionnaire; their weight and height were recorded. Participants were excluded if they were pregnant, used any nicotine product, had or were being treated for severe neuromuscular, cardiovascular, metabolic diseases, or cancer, were unable to take part in moderate physical activity, or had a body mass index (BMI) less than 25.0 or greater than 34.9 kg/m^2^. A BMI of at least 35 is required to be eligible for bariatric surgery but in an effort to recruit healthy individuals with limited chronic disease, it was strategized to use this same cut point as a maximum effort to recruit only those with limited co-morbidities for the study. In addition, the study dual energy X-ray absorptiometry (DXA) has a weight limit of 350 pounds with a limited scan window. This would make it difficult to get accurate body fat and muscle changes in individuals with a BMI greater than 35. Participants were eligible if they had medical conditions that were controlled by medication (e.g., hypertension, dyslipidemia). A total of 27 participants met the inclusion criteria and began the study. However, one participant withdrew from the study prior to pre-testing resulting in a final sample size of 26 participants (12% male).

### 2.2. Diet and Exercise Intervention

This study was a six week pilot study (diet and exercise) with small sample-size using a single group with no control arm of intervention and a pre-post testing design (clinicaltrials.gov #NCT04217850; 3 January 2020). Memberships to a wellness center at North Dakota State University were supplied to provide equipment access. Additionally, the wellness center supplied a private office to collect study-related weights, food diaries, and to offer 1:1 sessions with study subjects to assure compliance. Other measures, such as DXA and blood collection, were centralized in a biohazard/radiation safety approved lab on campus at the study university. Body composition (DXA), resting blood pressure, heart rate, waist-hip circumference, height, weight, and blood samples, were collected before the diet and exercise intervention (i.e., pre) and after six weeks of the intervention (i.e., post). All testing sessions were performed in the morning (i.e., from 05:30 to 09:00), and participants were instructed to fast for at least 8 h prior to testing. Blood pressure was evaluated manually with a sphygmometer. Height was measured to the nearest 0.1 cm using a stadiometer (Seca 213, Chino, CA, USA), and body mass was recorded on a portable scale (Denver Instrument DA-150, Arvada, CO, USA). The same scale was transported between testing locations and used for all weight measurements. Waist circumferences were measured between the iliac crest and the lowest rib—typically at the level of the umbilicus using a Gulick (Vancouver, WA, USA) measuring tape to the nearest 0.25 inch. A registered nurse or phlebotomist collected 6 mL of venous blood for miR analysis. Subjects provided written informed consent and the study was approved by the North Dakota State University Institutional Review Board (#EN20014). All procedures and methods were performed in accordance with the relevant guidelines and regulations.

### 2.3. Nutrition and Exercise Intervention

A registered dietitian (RD) guided each participant through dietary assessment, setting goals and follow-up evaluations at baseline, and every two to three weeks for a total of four visits, at weeks 0, 2, 4, and 6. This process followed the Academy of Nutrition and Dietetics Nutrition Care Process. As part of this process, participants were trained and instructed to complete a three day food log including one weekend or “off pattern” day prior to each meeting with a RD. Food logs were analyzed using ESHA Food Processor (Salem, OR, USA) at the point of collection by a team of research assistants and verified by a RD. Participants were weighed at each meeting not only to evaluate the efficacy of diet and exercise intervention, but also to ensure that participants were not losing weight too rapidly. The resting metabolic rate (RMR) determined energy expenditure and was based on DXA-derived Mifflin St. Jeor estimations [31]. A personalized nutrition plan was developed and updated along with body mass evaluations at each RD meeting. The principal goal was to restrict energy by 250 kcal/day—an agreed upon moving target set by participants and a RD. Dietary non-compliance was defined as exceeding the 250 kcal/day energy restriction by >200 kcal/day or demonstrating an inability or unwillingness to adhere to the agreed-upon individualized goals. Unwillingness to adhere to individualized goals included missing scheduled meetings with the dietitian, not filling out or inadequately filling out required food diary forms, and/or exceeding caloric restrictions on more than two consecutive days between time points 0 and 6 week. RDs reached out to participants to assure compliance and were on-call throughout the study if participants had any questions or concerns. No participants were considered non-compliant.

In addition to energy restrictions, participants performed supervised aerobic exercise 4–5 days per week for six weeks. At each exercise session at the campus wellness center, participants were prescribed goals to expend ≥250 kcals to further meet or exceed the total 500-kcal/day energy deficit. The intensity of the aerobic exercise sessions began at 55% of heart rate reserve and increased to 60% and 65% at weeks 4 and 6, respectively. The heart rate reserve was calculated based on resting heart rate and age-predicted heart rate max. The heart rate was measured using chest-worn heart rate monitors (Polar, Kempele, FL, USA). Trained graduate research assistants supervised the exercise implementation and were on-call throughout the study if subjects had any questions or concerns. Additional compliance for exercise and physical activity was measured with ActiGraph GT3X+ accelerometers (ActiGraph, Pensacola, FL, USA) worn at week 0 (before the intervention began) and week 6 of the intervention. Accelerometers were worn on the wrist during waking hours except for activities where the devices may get wet (e.g., bathing, swimming, etc.). A sleep log helped the subjects record the time and they removed the accelerometer for sleep and the time that they put the device on the following day upon waking. Accelerometers were set with epochs of 60 s, and data were collected at 80 Hz. For the physical activity assessment to be considered valid, the subjects had to have worn the accelerometer for at least four days including one weekend day over a seven-day collection period with a minimum of 10 h of wear time each day.

### 2.4. miR Analysis

Blood (6 mL) was drawn from fasting subjects by peripheral venipuncture into BD Vacutainer^®^ CPT™ (Becton Dickinson, Franklin Lakes, NJ, USA) with sodium citrate (Becton Dickinson, Franklin Lakes, NJ, USA). Serum was isolated from the blood by centrifugation at 4 °C and 2000× *g* for 10 min. The miRNA was then isolated from serum using a commercially available miRNA isolation kit (Qiagen, Germantown, MD, USA). Briefly, 600 µL serum sample was mixed with RPL buffer (Qiagen, Germantown, MD, USA), vortexed for about two seconds, and incubated at room temperature for three minutes. The sample was then mixed with RPP buffer (Qiagen, Germantown, MD, USA), vortexed for about twenty seconds, and incubated at room temperature for three minutes. Samples were centrifuged at 12,000× *g* for three minutes at room temperature to precipitate proteins. An equal amount of volume of isopropanol was added to the supernatant, and the sample was applied to the RNeasy UCP MinElute (Qiagen, Germantown, MD, USA), spin column where RNA binds to the membrane and other contaminants are washed away in subsequent wash steps with RWT buffer (Qiagen, Germantown, MD, USA), RPE buffer (Qiagen, Germantown, MD, USA), and 80% ethanol. Finally, the total RNA is eluted using 100 µL of low conductivity Tris Ethylenediaminetetraacetic acid (TE) buffer (conductivity = 5 μS/cm). The entire eluted sample in TE buffer (100 µL) was then used for miR detection.

miR detection was performed on the *iLluminate* device. Briefly, 100 µL sample was split into five 20-µL aliquots, and four samples were selectively hybridized (5 min at 95 °C and 55 min at room temperature) with commercially synthesized fluorescein- labeled complementary 22-mer DNA molecules (10 ng; Midland Certified Reagent, Midland, TX, USA). The following complementary DNA were used in the experiments.

1. miR-140-5p: 3′(FITC) CTACCATAGGGTAAAACCACTG5′
2. miR-935: 3′(FITC) GCGGTAGCGGAAGCGGTAACTGG5′
3. miR-99a: 3′(FITC)CACAAGATCGGATCTACGGGTT5′
4. miR-let-7b: 3′(FITC) AACCACACAACCTACTACCTCA5′

The fifth sample underwent hybridization without adding any fluorophore-labeled DNA to evaluate the background fluorescence. The miRNA with hybridized DNA, free miRNA, and free DNA, were analyzed using *iLluminate*. The detection and calculation of molarity values were discussed in our previous publication [35].

### 2.5. Body Composition

All participants were required to complete full-body DXA screening from prior to testing. Female participants provided a urine sample for pregnancy screening, and a positive test excluded the individual (Clinical Guard, Atlanta, GA, USA). Participants were asked to remove all metal and empty their pockets prior to the test. Researchers centered the participants relative to the midline of the DXA scanning platform and utilized an ankle strap to ensure symmetry and participant comfort. DXA scans were completed using a Lunar Prodigy scanner (GE Healthcare, Waukesha, WI, USA). Positioning was consistent for each scan with arms and hands in a neutral position and thumbs pointing upward to assure that proper imaging segmentation was obtained between the hips and hands. If a participant was outside the defined scanning window (given elevated BMI), then the internal estimation software was used for analysis. Data included total mass, tissue mass, fat mass, and lean mass.

### 2.6. Statistical Analyses

Statistical analyses were conducted in a stepwise fashion. Matched sample (pre-post) *t*-tests were used to assess whether study participants exhibited statistically significant reductions in body mass (fat, lean and total) and miR concentrations (i.e., molarities) over the course of the study. All matched sample *t*-tests operated under the specific null hypothesis of no mean change between pre- and post-intervention measurements.

Changes in miRs may vary between individuals who experience a substantial reduction in body mass versus those who do not experience substantial a substantial reduction [9]. Unfortunately, studies often define the cutoff between patients who achieve a “substantial” body mass reduction (a “high responder”) from those who fail to achieve a “substantial” mass reduction (a “low responder”) in an ad hoc fashion [9]. There is little guidance to define “high” and “low responders” especially in cases where sample sizes are small with a short study duration. This study adopted a conservative approach and used the sample median as a cutoff point. Individuals whose total body mass loss exceeded the sample median were deemed “responders” while all other participants were deemed “non-responders.” Repeated-sample ANOVA was used to determine whether statistically significant differences existed across key study variables. More specifically, for a specific miR, the test operated under the null hypothesis that the population means for responders (both pre- and post-intervention) and non-responders (both pre- and post-intervention) were equal.

A limitation of repeated-sample ANOVA is that it fails to control for the moderating effects of other factors that may influence a study participant’s body mass over the course of the study. To address this possibility, we estimated a reduced form, linear-in-parameters, difference-in-differences regression analysis with a common trend assumption [40]. A participant’s fat mass and lean mass were of primary interest, and thus the analysis was conducted twice: once for each type of mass. Pre- and post-intervention data were pooled in a non-matched fashion by constructing a binary variable (D) where a value of one indicated the post-intervention period. This binary variable interacted with the primary explanatory variables that consisted of concentrations of four miRs and a non-miR control. In this way, the coefficient estimates for the original miR variables represent pre-intervention levels and the coefficient estimates for the interaction between D and each miR variable represent a post-intervention effect.

Simple *t*-tests were applied to individual coefficient estimates to assess whether a significant relationship exists between miRs and a participant’s mass holding the study’s experimental design and the effects of all other control variables constant. Analogous assessments of the joint significance of these variables were implemented using an F-test. The overall model fit was assessed using an R-squared and adjusted R-squared metrics. The overall statistical significance of the model was assessed using the F-statistic. All hypothesis tests conducted in this study interpreted probability values less than 0.05 to be statistically significant. Statistical analyses were implemented using SAS, Version 9.4 (Statistical Software, Cary, NC, USA). Additional details about statistical analyses can be found in the supplementary information.

## 3. Results and Discussion

Pre-intervention descriptive statistics including age, height, weight, anthropometrics, blood pressure, and resting heart rate, on the full sample (*n* = 26) are shown in Table 1.

**Figure 3:**
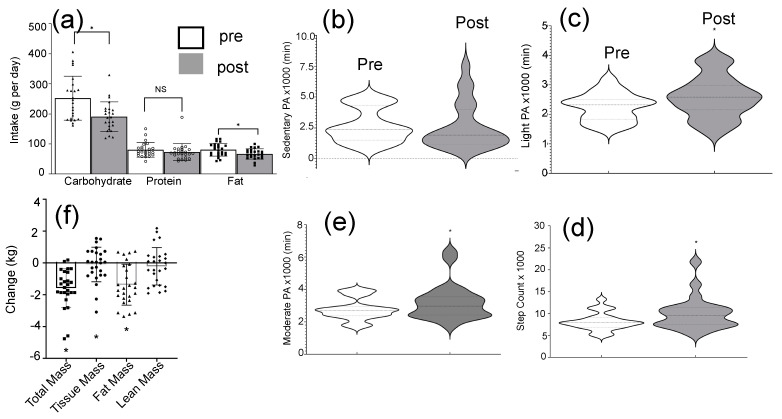
Results from diet and exercise intervention. In addition to blood sample collection, other important physiological properties were measured during the intervention. Additional physiological values were calculated and utilized to understand the meaning of miR results; (**a**) macronutrient intake (g per day). * *p* < 0.05, NS = non-significant. Physical activity (PA) compliance; (**b**) sedentary PA; (**c**) light PA; (**d**) moderate PA; (**e**) step counts. Note- no participants met thresholds for vigorous PA. * *p* < 0.05; and (**f**) variations of total, tissue, fat and lean masses of the subjects before and after the diet and exercise intervention * *p* < 0.05.

**Figure 4:**
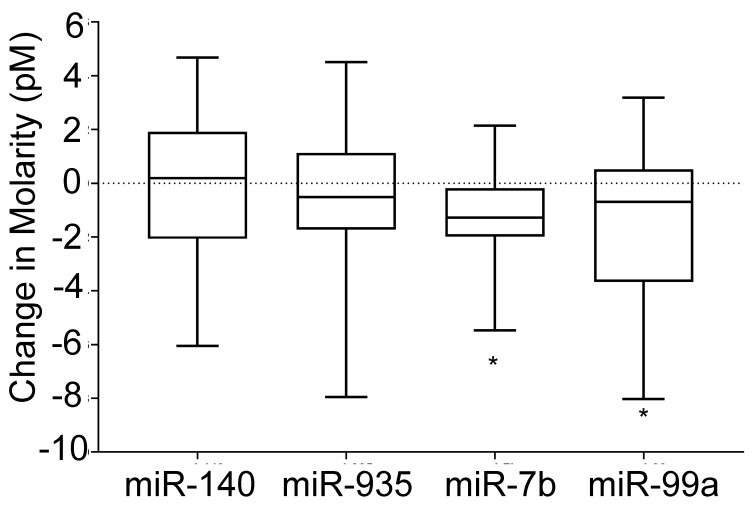
Variation of miRs-140, -935, -let-7b, and -99a, during the diet and exercise intervention. The change in molarity (post-pre) values were calculated. Molarity values were calculated using *iLluminate* for each miR target * *p* < 0.05.

Figure 3 illustrates the changes to energy intake, physical activity, and body mass of the subjects, during the intervention. Figure 3a shows the variation of micronutrient intake during the intervention. Carbohydrate intake (−24.4%, *p* = 0.001), and fat intake (−17.4%, *p* = 0.009) decreased, and protein intake was not significantly changed during the 6 week intervention period. Figure 3b–e show the physical activity of subjects during the intervention. Light (+14.4%, *p* = 0.040) and moderate (+16.6%) physical activity time as well as total steps counts (+21.9%, *p* = 0.002) significantly increased during the intervention. Figure 3f shows the changes (post-pre) to total body mass, tissue mass, and fat mass, during the intervention. Interestingly, the majority of subjects regardless of responders or non-responders reduced their fat mass. Figure 4 illustrates the variations (post-pre) of target miRs of the participants. In comparison, there were no changes (post-pre) in the median molarities of miR-140 and -935 following 6 weeks of diet and exercise (*p* > 0.05), but median values (post-pre) of miR let-7b and miR-99a were significantly reduced. The coefficients of variation (CV) for miRs-140, -935, -let-7b, and -99a were 8.3%, 5.4%, 11.3%, and 5.3%, respectively. Table 2 illustrates the changes in body weight, blood pressure, energy intake, and hip and waist dimensions. Interestingly, the total average values of energy intake were reduced by 22.6% and 18.2% of the non-responders and responders, respectively. Responders lost more total mass (*p* < 0.001), tissue mass (*p* < 0.001), and fat mass (*p* = 0.040) than non-responders (Table 2).

**Figure 5:**
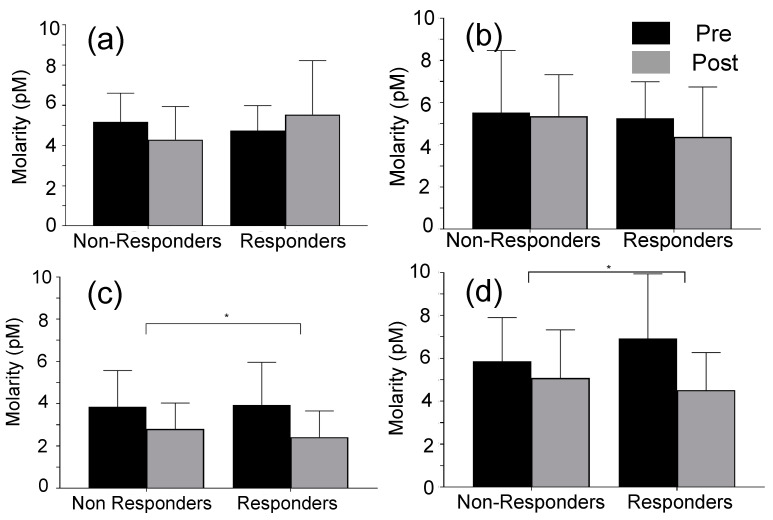
Variation of miRs levels of responders and non-responders to the diet and exercise intervention, * *p* < 0.05. The responders and non-responders were identified by calculating the % weight loss: (**a**) miR-140; (**b**) miR-935; (**c**) miR-let-7b; and (**d**) miR-99a.

In addition, responders lost a small amount of lean mass (48.3 to 47.7 kg). Figure 5 illustrates the variation (post-pre) of miR levels in both responders and non-responders. Average molarities of miR-140, -let7b and -99a were decreased during the intervention for non-responders, and there was no significant change to the average molarity of miR-935 for non-responders. Average molarities of miRs-let 7b, -935, and -99a, were decreased during the intervention for responders, and average miR-140 molarity was increased for responders. Table 3 shows the results from ordinary least squares regression analysis. The regression analyses predicted 91.7% and 87.6% of the variability in fat and lean body mass, respectively (*p* < 0.001). Restricted versions of these regressions that excluded all miR molarities yielded R-squared values of 84.8% and 78.8% for the fat mass and lean mass regressions, respectively. Thus, the miR covariates uniquely and jointly (and over and above the effects of the other model covariates) predicted 6.9% and 8.8% of the variation in fat mass and lean mass, respectively. The F-statistic indicates that this difference is significant for the fat mass regression (*p* < 0.05) but not significant for the lean mass regression (*p* = 0.055). At the level of the individual coefficients—and holding constant the effects of the other model covariates—miRs-140 and -935 significantly predicted fat loss (all *p* values < 0.05) (Table 3).

There were several findings: (1) *iLluminate* successfully detected the changes in miR levels in serum samples collected pre and post intervention; (2) in comparison to miR-7b and -99a, there was a high degree of variability for miR-935 and miR-140; (3) miRs let-7b and -99a declined with body mass loss; (4) after accounting for all other specified covariates, miRs-140, -935, let-7b, and -99a, collectively and uniquely predicted 6.9% and 8.8% of the variability in fat and lean body mass, respectively; and (5) miRs-140 and -935 had the greatest (statistically significant) predictive capability related to body fat mass. These observations may help future treatment of obesity and may have implications for understanding who succeeds and who does not in terms of weight loss with diet and exercise.

Similar work has been reported by Parr et al. who showed that miR-935 was higher in low responders (≤5% of weight loss) than high responders (≥10% weight loss) following 16 weeks of diet and exercise [10]. Given that our study was only 6 weeks long, we could not use the same reference cut-off points as this group for comparison (no subjects lost >10% body weight in 6 weeks). Our closest comparison is with a subset of the data in those who were deemed high responders (>5% of weight loss in 6 weeks, *n* = 2). These individuals showed a 64% decline (6.28 to 2.24 pM) in miR-935 over the intervention. The remaining sample (<5% of weight loss in 6 weeks, *n* = 24) only showed a 4% decline (5.31 to 5.08 pM) in miR-935. These data are consistent with Milagro et al., who also showed that miR-935 was increased in non-responders (<5% weight loss) versus responders (>5% weight loss) following 8 weeks of energy restriction alone [41]. Further analysis using a median split of the sample showed that non-responders (lowest 50% of sample, *n* = 13) had a decline of miR-935 of only 3%, while responders (upper 50% of sample, *n* = 13) had a decline of 16%. miR-935 appears to be related to the activity of transcription factors and nuclear receptors associated with fat oxidation and the regulation of energy expenditure. Overall, these data show similar trends to Parr et al. [10] and Milagro et al. [41] with differences in the pre-post intervention suggesting higher concentrations of miR-935 in circulation in those who struggle with weight loss; there was a lower concentration in those who have successfully lost weight.

Parr et al. [10] also showed that circulating miR-140 increased in low responders (≤5% of weight loss) versus high responders (≥10% weight loss) following 16 weeks of diet and exercise. Higher levels were also identified in other species (miR-142) in obese individuals [42]. Here, miR-140 did not show an upregulation in either non-responders (lowest 50% of sample, *n* = 13) or responders (upper 50% of sample, *n* = 13). miR-140 appears to be related to insulin signaling and glucose transport in insulin-sensitive tissues [10]. We speculate that the duration of the exercise and nutrition intervention was not sufficiently long to see changes in circulating miR-140 levels. Together, miR-935 and -140 significantly predicted fat loss at the level of the individual regression coefficients, which indicates their value in body weight management.

Perhaps the most interesting finding of our study was the significant decline in miR-let-7b and 99a following 6 weeks of diet and exercise. As discussed above, these changes could be due to fat dissolution or glucose intolerance during the intervention. Surprisingly, down-regulation of miRs let-7b and -99a occurred in both responders and non-responders. Additional studies may be needed to determine the exact biological pathways for observed miR level changes. In addition to weight loss, these two miRs have used in cancer detection [43,44], but each showed consistent decreases in concentration with weight loss. miR-let-7b has been previously shown to be downregulated in acute myocardial infarction in myocardial tissues in a rat model [43]. miR-99a is also implicated in squamous cell carcinoma [44]. Here, miRs let-7b and -99a join others (miRs 122, 193a-5p, 16-1) that have also been reduced with weight loss [42]. However, in our regression analysis, miRs let-7b and -99a did not contribute strong or significant estimates to the model, thus minimizing their role in predicting weight loss. In addition, the role of miRs in adipose tissue physiology and obesity related metabolic disorders have been summarized in recently published literature [45,46,47].

Some limitations of the study should be noted. Participants self-reported energy intake and expenditure through three-day food logs and exercise logs. While participants were coached and monitored to elicit an average deficit of 500 kcal/day, actual compliance could not be guaranteed. Other limitation of the study is the lack of discussion about miRs expression variation with glucose and triglyceride levels. The longitudinal nature of body mass reduction relies on significant units of time, and thus further research will benefit from a greater study duration. While perfect compliance is impossible to guarantee outside of a metabolic ward, studies using free-living experimental designs provide strong external validity to stressors impacting individuals seeking to lose weight.

## 4. Conclusions

Prior to detection of miRs using *iLluminate*, miR isolation and hybridization of target miRs with fluorophore-labeled complementary DNA hybridization still require manual steps that may need some technical expertise. We are currently working on developing miR isolation and miRNA-DNA hybridization methods that can be performed at the point-of-care. Moreover, electric field-based miRNA and DNA hybridization methods can easily be integrated with other assay steps in the device. The miR expression levels obtained from *iLluminate* were consistent with previously reported miR levels. Therefore, *iLluminate* can be used to accurately detect miRs in clinical samples. There was considerable variability in body mass loss with the energy deficit facilitated by the prescribed diet and exercise in overweight and obese adults. Within a relatively short duration (6 weeks) of body mass loss, miRs-140 and -935 provided predictive capability for body fat adaptability when controlling for other external factors. These miRs may still be useful to monitor future larger clinical trials when treating obesity-related conditions to ascertain if their baseline circulation influences the success of body mass and fat loss. Furthermore, miRs let-7b and -99a appear to be biomarkers that mirror changes in body mass loss following a short-term diet and exercise intervention.

In conclusion, this work demonstrated that *iLluminate* could detect multiple miRs from clinical samples. If the detection steps are automated, then *iLluminate* can potentially be used at the point-of-care. The miR biomarkers that we selected for this study were sensitive to weight loss, but more work is needed to determine the impact of demographic factors (e.g., age, sex, and ethnicity). In addition, the ability of these biomarkers to predict the long-term effects of weight-loss is still unknown. Further studies are ongoing.

## Figures and Tables

**Table 1:** Description of participants.

	Mean ± SD
Age (years)	39.13 ± 13.03
Height (cm)	168.73 ± 7.71
Body Mass (kg)	84.43 ± 11.35
BMI (kg/m^2^)	29.57 ± 2.53
Hip Circumference (cm)	110.81 ± 4.93
Waist Circumference (cm)	89.46 ± 8.43
Resting HR (bpm)	72.46 ± 9.64
Resting Systolic BP (mmHg)	126.85 ± 9.60
Resting Diastolic BP (mmHg)	81.46 ± 9.49

The sample was 26 (23 females, 3 males). Data are presented as mean ± SD. Body Mass Index (BMI), Heart Rate (HR), Blood Pressure (BP).

**Table 2:** Responders and non-responders to the 6 week diet and exercise intervention.

	Non-Responders (*n* = 13)		Responders (*n* = 13)		*p* Values	
	Pre	Post	Pre	Post	Time	Interaction
Tissue mass (kg)	84.9 ± 9.8	84.2 ± 9.7	78.8 ± 11.5	76.3 ± 11.9	*p* < 0.001	*p* < 0.001
Fat mass (kg)	35.1 ± 6.5	34.3 ± 6.5	30.5 ± 6.6	28.6 ± 6.8	*p* < 0.001	*p* = 0.040
Lean mass (kg)	49.7 ± 7.7	49.9 ± 7.3	48.3 ± 8.0	47.7 ± 7.9	*p* = 0.354	*p* = 0.126
Total mass (kg)	87.6 ± 10.2	86.9 ± 10.1	81.3 ± 11.8	78.8 ± 12.2	*p* < 0.001	*p* < 0.001
Systolic blood pressure (mmHg)	130.3 ± 9.2	120.9 ± 7.0	123.4 ± 9.1	118.5 ± 11.7	*p* = 0.002	*p* = 0.287
Diastolic blood pressure (mmHg)	85.4 ± 9.4	77.3 ± 6.6	77.5 ± 8.1	73.1 ± 8.5	*p* = 0.005	*p* = 0.376
Resting heart rate (bpm)	76.8 ± 9.6	70.2 ± 12.1	68.2 ± 7.9	62.0 ± 9.0	*p* < 0.001	*p* = 0.875
Waist circumference (cm)	92.2 ± 7.9	90.2 ± 9.9	86.8 ± 8.4	82.5 ± 9.4	*p* = 0.001	*p* = 0.180
Hip circumference (cm)	112.1 ± 5.5	109.8 ± 5.9	109.5 ± 4.1	105.9 ± 4.2	*p* < 0.001	*p* = 0.057
Daily energy intake (kcal)	2257 ± 592	1748 ± 441	1932 ± 392	1581 ± 258	*p* < 0.001	*p* = 0.393

Data are mean ± SD.

**Table 3:** Ordinary Least Squares regression analysis.

				*t*-Test				*t*-Test
Regressor	Estimate	St. Error	*t*-Test	Prob.	Estimate	St. Error	*t*-Test	Prob.
***Regression Intercept & Pre-Post***								
Intercept	−46.406	18.634	−2.490	0.019	−59.656	24.675	−2.420	0.022
Post-Intervention Indicator	1.082	5.250	0.210	0.838	−5.565	6.953	−0.800	0.430
***Subject Characteristics***								
Systolic BP (mmHg)	0.006	0.069	0.080	0.935	0.072	0.091	0.790	0.435
Diastolic BP (mmHg)	0.029	0.074	0.390	0.697	−0.091	0.098	−0.930	0.359
Resting Heart Rate (bt/min)	0.098	0.055	1.760	0.088	−0.061	0.073	−0.830	0.416
Waist Measurement (cm)	0.359	0.083	4.310	<0.001	0.002	0.110	0.020	0.983
Hip Measurement (cm)	0.713	0.095	7.470	<0.001	0.211	0.126	1.670	0.105
Energy Expenditure (kcal)	−0.0004	0.001	−0.650	0.521	−0.001	0.001	−0.790	0.436
Energy Intake from Diet (kcal)	0.00001	0.001	0.010	0.990	−0.001	0.002	−0.930	0.358
Participant Age (yr)	−0.053	0.047	−1.110	0.276	−0.109	0.063	−1.740	0.092
Height (cm)	−0.252	0.109	−2.300	0.028	0.597	0.145	4.120	<0.001
Gender	−0.712	2.002	−0.360	0.725	6.584	2.651	2.480	0.019
***miRs Molarities***								
miR 140	1.848	0.593	3.120	0.004	−1.750	0.785	−2.230	0.033
miR 140 ^1^	−1.940	0.668	−2.900	0.007	1.430	0.885	1.620	0.117
miR 935	−1.037	0.319	−3.260	0.003	0.334	0.422	0.790	0.435
miR 935 ^1^	1.905	0.435	4.380	<0.001	−1.393	0.576	−2.420	0.022
miR Let-7b	−0.361	0.377	−0.960	0.345	0.746	0.499	1.490	0.145
miR Let-7b ^1^	0.498	0.597	0.830	0.411	0.315	0.791	0.400	0.694
miR 99a	0.158	0.263	0.600	0.553	−0.337	0.349	−0.970	0.341
miR 99a ^1^	−0.173	0.370	−0.470	0.643	0.562	0.490	1.150	0.260
miR Control	−0.021	1.483	−0.010	0.989	1.354	1.964	0.690	0.496
miR Control ^1^	3.119	11.655	0.270	0.791	7.773	15.434	0.500	0.618
R-Square	0.917				0.876			
Adjusted R-Square	0.860				0.789			
F[21,30]-Statistic of Overall Model Fit	15.850			<0.001	10.060			<0.001
F[10,30]-Statistic, Joint Significance of All miR Coefficients	2.499			0.026	2.117			0.055
F[5,30]-Statistic, Joint Significance of All Pre-Intervention miR Coefficients	2.713			0.017	2.093			0.058
F[5,30]-Statistic, Joint Significance of All Post Intervention miR Coefficients	4.643			0.001	1.534			0.176
Number of Observations	52				52			

^1^ Post intervention.

## Data Availability

The datasets generated during and/or analyzed during the current study are available from the corresponding author on reasonable request.

## Supplementary Materials

The following supporting information can be downloaded at: https://www.mdpi.com/article/10.3390/s22186758/s1.
