# Supplementary material for: A Miniaturized MicroRNA Sensor Identifies Targets Associated with Weight Loss in a Diet and Exercise Intervention among Healthy Overweight Individuals
Source: Sensors (Basel). 2022 Sep 7;22(18):6758. doi: 10.3390/s22186758 (PMC9505139; doi:10.3390/s22186758)
Supplement: Supplementary file 1 [file sensors-22-06758-s001.zip › sensors-1821237-supplementary.pdf]

Statistical Analyses

The statistical analysis operated under the general null hypothesis that no statistical relationship existed between c-miRs and a participant's body mass (total, tissue, fat or lean), whether these variables were measured before the intervention, after the intervention, or as changes across time. All hypothesis tests conducted in this study interpreted probability values less than 0.05 as statistically significant. Statistical analyses were implemented using SAS, Version 9.4 (Statistical Software, Cary, NC, USA).

Assessment of the study's general null hypothesis proceeded in a stepwise fashion. First, matched sample (i.e., pre-post) t-tests were used to assess whether study participants exhibited statistically significant reductions in body mass (fat, lean and total) and c-miR concentrations (i.e., molarities) over the course of the study. Consistent with the general null hypothesis, all matched sample t-tests operated under the specific null hypothesis of no mean change between pre- and post-intervention measurements (i.e., the pre-intervention mean equals its corresponding post-intervention mean).

The literature suggests that changes in c-miRs may vary between individuals who experience a substantial reduction in body mass, versus those who do not experience substantial a substantial reduction [9]. Unfortunately, studies often define the cutoff between patients who achieve a "substantial" reduction in body mass (or a "high responder") from those who fail to achieve a "substantial" mass reduction (or a "low responder") in an ad hoc fashion [9]. With little guidance on how to define "high" and "low responders" (especially in cases - such as the current study - where sample sizes are relatively small and the duration was only 6 weeks), this study adopted a conservative approach and used the sample median as a cutoff point. Those individuals whose total body mass loss exceeded the sample median were deemed "Responders", while those participants whose loss was at or below the sample median were deemed "Non-responders". Repeated samples ANOVA was used to determine whether statistically significant differences existed across key study variables (including, but not limited to, c-miR concentrations). More specifically, for a given variable of interest (i.e., a specific c-miR), the test operated under the null hypothesis that the population means for responders (both pre- and post-intervention), and non-responders (both pre- and post-intervention) were equal.

A limitation of repeated samples ANOVA is that it fails to control for the moderating effects of other factors that may exogenously influence a study participant's body mass over the course of the study. To address this possibility, we estimated a reduced form, linear-in-parameters, difference-in-differences regression analysis with a common trend assumption [36]. More specifically, ordinary least squares regression was used to estimate a (reduced form, linear-in-parameters) regression of the following form:

$$Wt_{it} = \alpha_0 + \alpha_1 D_t + \sum_{j=1}^{J} \beta_j X_{it}^j + \sum_{j=1}^{J} \gamma_j D_t X_{it}^j + \sum_{k=1}^{K} \delta_k Z_{it}^k + \sum_{l=1}^{L} \varphi_L Q_i^L + u_{it} \quad (1)$$

where i = 1,…,n is the number of study participants; t indicates the pre (t=1) and post (t=2) time periods; Wt is a measure of participant body mass; D is a binary variable that assigns a value of 1 to the post-intervention period and a value of 0 to the pre-intervention period. X^j represents a series of j = 1,…J cmiRs and a non-cmiR control; Z^k represents a series of k=1,…,K control variables that vary by participant and time (heart rate, waist measurement, etc.), Q^l is a series of l=1,…,L variables that vary by participant but not over time (participant gender, height, etc.), the αs, βs, γs, δs, and φs are parameters to be estimated, and u is an error term with the usual assumptions (independent and identically distributed, etc.). Because a participant's fat mass, and lean mass were of interest, the regression analysis was conducted two times, once for each type of mass. Regression analyses utilizing total mass as the dependent variable were also conducted. Due to space constraints,

these results were not reported in this manuscript, but are available in the manuscript's supporting documents.

Under this specification, pre- and post-intervention data were pooled in a non-matched fashion. This binary variable was interacted with the primary explanatory variables, which consisted of the concentrations of four c-miRs and a non-c-miR control. In this way, the coefficient estimates for the original c-miR variables (the β_js) represent pre-intervention levels and the coefficient estimates for the interaction between D and each c-miR variable (the γjs) represent a post-intervention effect. Simple t-tests (operating under the null hypothesis that a given population parameter is zero) were applied to each individual coefficient estimates to assess whether a significant relationship exists between c-miRs and a participant's mass, holding constant the study's experimental design and the effects of all other control variables included in the regression. The test has n-p degrees of freedom, where n is the sample size and p is the number of coefficient estimates in the regression including the intercept. Analogous assessments of the joint significance of these variables were implemented using an F-test with v and n-v degrees of freedom, where v is the number of restrictions in the null hypothesis. Overall model fit was assessed using the R-square and adjusted R-square metrics, and the overall statistical significance of the model was assessed using the F-statistic with p-1 and n-p, degrees of freedom, respectively.
